# Significantly improved outcomes – both in retention and CGI scores - using Long Acting Buprenorphine (LAB-Buvidal) versus treatment as usual for Opioid Dependence in Wales during the Pandemic

**DOI:** 10.1192/j.eurpsy.2022.2106

**Published:** 2022-09-01

**Authors:** J. Melichar, L. Pearson, E. Richards, A. Lindsay, R. Greenwood

**Affiliations:** 1 Cardiff and Vale University Health Board, Community Addiction Unit, Cardiff, United Kingdom; 2 Cardiff and Vale University Health Board, Community Drugs And Alcohol Services, Cardiff, United Kingdom; 3 University Hospitals Bristol and Weston, Research Design Service, Bristol, United Kingdom

**Keywords:** buvidal, long acting, Addiction, buprenorphine

## Abstract

**Introduction:**

We have been using LAB (Buvidal) in Cardiff after its pandemic use was funded by Welsh Government.

**Objectives:**

We wished to review the benefits of introducing LAB (Buvidal) into treatment during the pandemic.

**Methods:**

This service development review of the first 73 patients treated with LAB (24mg/96mg rapid titration Welsh protocol) was analysed using Kaplan-Meier survival curves.

**Results:**

43 (58%) male, 30 (41%) female. <25years=1, 38 (52%) aged 25-40, 34 (47%) 40-55. Prior to LAB 14% (10 people) using Espranor, 8% (6) Buprenorphine, 28% (20) Methadone. 50% (36) illicit opiates (mainly Heroin). We had continuous data for patients for up to 9 months of LAB. Two stopped for non-discontinuation reasons: One wanted to detox, one died of natural causes (LAB-unrelated). Both were excluded from discontinuation rate analysis. 55 people have data for over a month. Of these, 11 discontinued treatment. 80% remained on LAB for 1 month or more [95%CI 67-90%]. Kaplan-Meier plots showed similar discontinuation rates when comparing different OST programmes or none prior to LAB, and comparing by age, sex and initial illness severity (CGI severity). These rates all far exceeded data for traditional OST. CGI scores dramatically improved, even at one week. By month 2 all scores “much improved” or “very much improved”.

**Conclusions:**

Buvidal (LAB) has 80% retention rates, regardless of underlying prescribed/illicit opioid /demographics. The commonly held belief that those on heroin are further from Recovery than those more stable on OAD may be incorrect. LAB may be a more acceptable and useful first line therapy that other OSTs

**Disclosure:**

Dr Melichar has provided consultancy work, presentations, training and chaired panel discussions for all the companies in this area in the UK and some outside the UK. Recent work includes Althea (UK), Britannia (UK), Camurus (UK and Global), Martindale (U

